# Phenotypic Variation and Genetic Architecture for Photosynthesis and Water Use Efficiency in Soybean (*Glycine max* L. Merr)

**DOI:** 10.3389/fpls.2019.00680

**Published:** 2019-05-24

**Authors:** Miguel Angel Lopez, Alencar Xavier, Katy Martin Rainey

**Affiliations:** ^1^Department of Agronomy, Purdue University, West Lafayette, IN, United States; ^2^Corteva Agriscience, Johnston, IA, United States

**Keywords:** gas exchange, yield, soybean, genomic prediction, efficiency, biomass

## Abstract

Photosynthesis (*A*) and intrinsic water use efficiency (*WUE*) are physiological traits directly influencing biomass production, conversion efficiency, and grain yield. Though the influence of physiological process on yield is widely known, studies assessing improvement strategies are rare due to laborious phenotyping and specialized equipment needs. This is one of the first studies to assess the genetic architecture underlying *A* and intrinsic *WUE*, as well as to evaluate the feasibility of implementing genomic prediction. A panel of 383 soybean recombinant inbred lines were evaluated in a multi-environment yield trial that included measurements of *A* and intrinsic *WUE*, using an infrared gas analyzer during R4–R5 growth stages. Genetic variability was found to support the possibility of genetic improvement through breeding. High genetic correlation between grain yield (*GY*) and *A* (0.80) was observed, suggesting increases in *GY* can be achieved through the improvement of *A*. Genome-wide association analysis revealed quantitative trait loci (QTLs) for these physiological traits. Cross-validation studies indicated high predictive ability (>0.65) for the implementation of genomic prediction as a viable strategy to improve physiological efficiency while reducing field phenotyping. This work provides core knowledge to develop new soybean cultivars with enhanced photosynthesis and water use efficiency through conventional breeding and genomic techniques.

## Introduction

Since the early 1900s, soybean yields have increased steadily (Hartwig, 1973; Specht et al., 1999; Suhre et al., 2014) and the rate of annual increases is estimated as 22–27 kg ha^-1^ yr^-1^ (Specht et al., 1999; USDA-ERS, 2011; Fox et al., 2013; Koester, 2014). Considering the gap between the current and the maximum efficiency of light conversion into biomass (Melis, 2009; Zhu et al., 2010), a higher rate of gain may be achievable. Doubling the efficiency of converting solar radiation into biomass is still theoretically possible for soybean, as current values of radiation use efficiency range from 2.3 to 4.3% and the theoretical maximum was estimated to be 9.4% (Beadle et al., 1987; Zhu et al., 2010). Improving the conversion of solar radiation into biomass and yield requires the optimization of physiological and biochemical processes linked to CO_2_ uptake and reduction, water loss, CO_2_ uptake – water lost relationship, and nitrogen assimilation. Some of these correspond to the gas exchange dynamic, specifically photosynthesis (*A*) and water use efficiency (*WUE*). Although a positive relationship between photosynthesis and yield is not always observed (Long et al., 2006), a positive correlation between yield and photosynthesis has been found in soybean (Ainsworth et al., 2012). This positive association is also documented for other crops like rice, where Ohsumi et al. (2007) and Peng et al. (2008) reported higher photosynthetic rates and improved physiological traits as significant contributors for high yielding cultivars. Similar results were presented by Tollenaar (1991) and Duvick (2005) in maize, and Fischer et al. (1998) and Xiao et al. (2012) in wheat.

Although gas exchange parameters in soybean are well-documented using small panels (Bruns, 2014; Gai et al., 2017; Li S.Y. et al., 2017; Ort et al., 2017), studies of larger panels are still needed to make genetic inferences. In this study, photosynthesis and intrinsic water use efficiency as valuable parameters for breeding purposes in soybean are explored through a set of field experiments where a relatively large and diverse panel was evaluated. This research focuses on determining the natural diversity of *A* and *WUE* in soybean. Likewise, the genetic architecture of these traits is revealed through a genome wide association approach. Finally, the viability of implementing genomic prediction is assessed.

## Materials and Methods

### Plant Materials

In this study, 383 recombinant inbred lines (RIL) from 32 families (12 RILs per family) coming from a subset of the Soybean Nested Association Mapping (SoyNAM) panel (Diers et al., 2018; Xavier et al., 2018) were assessed. Lines were selected with the goal of creating a panel constrained for maturity while retaining phenotypic variance for yield and other traits. Selection of the RILs was based on Best Linear Unbiased predictors (BLUP) for maturity, measured as the number of days from planting to physiological maturity, corresponding to soybean growth stage R8 (Fehr and Caviness, 1977), and grain yield calculated from field experiments from Indiana and Illinois during the years 2011 and 2014 (Xavier, 2016). The RILs selected for the panel were in the maturity range of ±2 days, while the yield varied from 3,088 to 4,396 kg ha^-1^ (Supplementary Figure S1). The panel includes lines from three classes of families: 16 from elite parents, 12 with diverse pedigrees, and four that are high-yielding under drought conditions (Supplementary Table S1). The NAM hub parent for the families is cultivar IA3023. The details about families are available in SoyBase through the website^1^ and the full list of RILs is also presented here in Supplementary Table S2.

### Field Design

Experiments were grown in one location in 2017 and two locations in 2018. An alpha lattice incomplete block design with 383 RILs, two complete replications and 32 incomplete blocks per replication was planted at the Purdue University Agronomy Center for Research and Education – ACRE (40°28′20.5″N 86°59′32.3″W) in 2017 (ACRE_2017). Experimental units corresponded to six rows plots (0.76 m × 3.35 m) with a seeding rate of 35 seeds m^-2^. In 2017, 66 RILs were discarded as consequence of non-uniform emergence. In 2018, the same experimental design was planted in two locations: ACRE (ACRE_2018) and Romney, IN (40°14′59.1″N 86°52′49.4″W; RMN_2018), with data collected from 382 and 368 RILs, respectively. Soil types included Chalmers silty clay loam (Typic Endoaquolls) and Raub-Brenton complex (Aquic Argiudolls) for ACRE and Drummer soils (Typic Endoaquolls) for Romney (NRCS, 2018). High natural soil fertility confirmed through the soil analysis (Supplementary Table S3), along with the crop management ensured adequate nutritional status during the growing season. Mean precipitation reached 132, 130, and 91 mm/month for ACRE_2017, ACRE_2018, and RMN_2018, respectively (iClimate, 2019). Absence of water stress throughout the growing season and in particular during the measurements period was confirmed through the water balance (Supplementary Tables S4–S6). FAO 56 guide was followed as theoretical framework for the water balance (Allen et al., 1998), while the specific information about field capacity, permanent wilting point, and bulk specific density was retrieved from Wiersma (1984). Reference evapotranspiration was computed through Hargreaves et al. (1985) equation and the crop coefficients (*K*_c_) were obtained from Al-Kaisi (2000).

### Field Phenotyping

Gas exchange is a biological process influenced by several environmental factors including photosynthetic active radiation (PAR), CO_2_ concentration, water and nitrogen status, and temperature (Taiz et al., 2014). To account for most of these sources of variation and obtain comparable measurement from all plots, a highly controlled gas exchange protocol was implemented using a portable photosynthesis system (LI-COR 6400XT, LI-COR, Lincoln, NE). An initial light response survey using the rapid protocol proposed by LI-COR (2012) was carried out in random plots to establish the minimum amount of PAR required to get stable, constant flat assimilation rates (Supplementary Figure S2). The consistency of this PAR value (1,600 μmol photons m^-2^ s^-1^) was confirmed in different random selected cultivars and subsequently set as constant for the measurements. The LED light source within the 6 cm^2^ chamber was used. To control other variables affecting the gas exchange, CO_2_ concentration and temperature were also set as constant at 400 μmol mol^-1^ and 25°C, respectively. The relative humidity was restricted to 75 ± 10%. To avoid non-adapted reading as consequence of significant differences between the external environment and the chamber, each leaf was previously adapted for at least three minutes or until getting stable readings. Outlier determination following the criterion 1.5 × interquantile range (IQR) were carried out and atypical values caused by non-adapted leaves were eliminated. Normality in the data set was confirmed through histograms (Supplementary Figure S3).

The gas exchange parameters were measured before the seed filling phenological period, from late R4 and early R5 (Fehr and Caviness, 1977), in the third uppermost fully developed leaf, in three representative plants from each experimental unit from a complete replication. This specific phenological stage was chosen based on literature reports of maximum rates of crop photosynthesis, crop growth, and pod production (Egli and Bruening, 2002; Board and Kahlon, 2012). In addition, natural or induced variation in the photosynthetic rate during this period directly influences the yield components number of pods and number of seeds (Egli, 2010). To confirm developmental synchronization among cultivars, R5 was scored in the panel obtaining ranges of 6, 5, and 9 days for ACRE_2017, ACRE_2018, and RMN_2018, respectively. Four portable photosynthesis systems previously calibrated with equal configurations were used in daily sampling protocol spanning approximately 7 h (10:00 h–5:00 h). Sampling occurred over a period of less than 6 days at each location. Negligible influence of diurnal time on the readings was confirmed plotting the reading versus daily time (Supplementary Figure S4). This protocol evaluates the maximum photosynthetic capability and their associated intrinsic water use efficiency (ratio photosynthesis/stomatal conductance – *gs*) in field under comparable conditions. Finally, the rows four and five of each plot were mechanically harvested and weighted for yield calculation. Moisture for grain yield (*GY*) was standardized to 13% and extrapolated to hectare.

### Genomic Information

The complete SoyNAM panel was genotyped through an illumina soybean array designed specifically for the NAM population, the SoyNAM6K BeadChip SNP, with 5,305 single nucleotide polymorphism (SNP) markers (Song et al., 2017). These markers were originally identified using the genome sequences of the founder parents (41). Besides genome sequence, the SoyNAM founders parents were also evaluated with the soySNP50K Beadchip (Song et al., 2013) detecting 42,509 SNP markers. Using the framework of the mapped SoyNAM6K markers and the software *finhap f90* (VanRaden et al., 2015), the segregating SoySNP50K markers were projected onto the SoyNAM RILs. “Williams 82” reference genome (Wm82.a2.v1) positions in pair bases (*pb*) were used. Quality control for minor allele frequency (MAF < 0.15) (Jarquín et al., 2014; Xavier et al., 2016) was performed in the projected SNPs data set ending up with 23,119 SNPs, which are used as genotypic information. The original allele frequency plot as well as the representation of each RIL in the principal component plot were also explored to discard unusual patterns (Supplementary Figure S5).

### Statistical Model and Data Analyses

Data collected were consolidated and analyzed using the mixed model approach in the software R through the package “lme4” (Bates et al., 2015). Sources of variation were: environment (combination year × location), block, and RIL, with the covariate of equipment (1). Though equipment of the same model and configuration were used, differences due to a particular analyzer were removed with this covariate. The model implemented is:

(1) $$Y_{\text{ijk}}\text{=}\mu \text{+}\alpha_{\text{i}}\text{+}\beta_{\text{j}}\text{+}\gamma_{\text{k}}\text{+}{\left(\beta \gamma \right)}_{\text{jk}}\text{+}\delta_{\text{l}}\text{+}e_{\text{ijk}}$$

where *Y* is the vector of phenotypes measured with the *i*th equipment in the *j*th environment into the *k*th block, μ is the intercept, α accounts for the effect of the covariate equipment, β corresponds to the effects of environment, δ accounts for the block effect, βγ corresponds to the interaction environment × block, δ accounts for the genetic effect, and *e* controls the error. The covariate was treated as a fixed effect while the other sources of variation were considered as random. Given the limitations in humidity control offered by the LI-COR 6400XT and the range set in this research for relative humidity, leaf vapor pressure deficit (VPD_L_) reported by the equipment was also used as covariate in the model fitted for *WUE*.

### Association Analysis

A genome wide association analysis under the empirical Bayesian framework was performed using the R package “NAM” (Xavier et al., 2015). In each case, BLUPs from Eq. 1 was treated as phenotypes, while the set of 23,119 projected SNPs was used as genotypes. Population structure was accounted for under the argument *fam* in the function *gwas2*. The base model for the genome scanning is described by:

(2) y=μ+Zu+g+e

where *y* corresponds to the BLUPs values, *Zu* is the incidence matrix of haplotypes generated from marker data, μ is the vector of regression coefficients of within-family marker effects, *g* corresponds to the polygenic coefficients accounting for population structure, and *e* is the vector of residuals. Statistical significance of each marker was calculated using the likelihood ratio test (LRT) between a full model including the marker, *Zu*, and a reduced model without marker. The association between markers and traits was evaluated using a corrected *p*-value threshold of 0.0002. This threshold corresponds to the *p*-value of 0.01 divided by the number of unique segments (58), estimated as the number of significant eigenvalues computed from the spectral decomposition of the genetic relationship matrix. The R package CM-plot was used to create the Manhattan plots (LiLin, 2018). To discard confounding effects, the signals were contrasted with known quantitative trait loci (QTLs) for maturity genes (Zhang et al., 2014). The exploration for potential candidates genes was carried out in the range on the linkage disequilibrium (LD) reported for each chromosome (Wen et al., 2015).

### Genetic, Additive Variances, and Genetic Correlation

Broad-sense heritability (*H*) on an entry mean basis and plot basis was calculated from model (1) using the variance components from the mixed model in the Eq. 1. Heritability on an entry mean basis was calculated through the equation below (Nyquist, 1991; Piepho and Möhring, 2007).

(3) $$H=\frac{Vg}{Vg+\frac{Ve}{r}}$$

where *H* corresponds to broad sense heritability on mean entry basis, *Vg* is the genetic variance, *Ve* is the variance of error, and *r* is the number of replications. Heritability on a plot basis followed the same equation, but the variance of error was not weighted into the number of replications. Narrow sense heritability (*h^2^*) was also calculated from different whole-genome regressions using the expectation-maximization restricted maximum likelihood method from the “NAM” package (Xavier et al., 2015). For each regression, a different subset of SNPs was considered based on the -log *p*-values from the association analysis (Table 1). Subsets of genomic data with markers that displayed -log *p*-values higher than 0.0, 0.5, 1.0, 1.5, and 2.0 to the target traits were considered.

**Table 1:** Number of SNPs for each data set created using as discrimination parameter the –log *p*-value from the association analysis study.

Trait	-log *p*-value > 0	-log *p*-value > 0.5	-log *p*-value > 1.0	-log *p*-value > 1.5	-log *p*-value > 2.0
*A*	7,025	5,408	2,226	714	220
*WUE*	8,043	6,171	2,619	845	230

To determine the genetic correlations, a multivariate mixed model using the restricted maximum likelihood (reml) approach was solved using as response variable a matrix with the BLUPs values for *A, WUE*, and *GY* from Eq. 1. A genetic relationship matrix calculated from the full data set of SNPs was included in the model to account for the genetic effect. The function reml from the NAM package (Xavier et al., 2017a) was used. However, correlation involving *WUE* are not reported here since it is a derivate variable from photosynthesis and stomatal conductance (*gs*).

### Genomic Prediction

Using the SNPs above the significance threshold of -log *p*-values from 0 to 2 from the genomic information already described, a set of whole-genome regressions were computed using the Bayesian framework (de los Campos et al., 2013). Seven whole-genome regression methods were fitted via Markov chain Monte Carlo (MCMC) implemented in the R package bWGR (Xavier et al., 2018): *BayesA, BayesB, BayesC*, BLASSO (*BayesL*), Bayes ridge regression (*BayesRR*), *BayesCpi* and *BayesDpi* (Habier et al., 2011). Likewise, seven methods fitted via expectation maximization (EM): BayesA (*emBA*), BayesB (*emBB*), BayesC (*emBC*), BLASSO (*emBL*), BLASSO2 (*emDE*), maximum likelihood (*emML*), and Bayesian ridge regression (*emRR*) were also fitted. Five-fold cross-validation was implemented splitting the data set randomly in proportions 80 (training): 20 (validation) each time. Correlation coefficients between measured and predicted breeding values in the validation set were calculated each time. Function *mcmcCV* and *emCV* from the R package bWGR were used to perform the cross-validations.

## Results

### Phenotypic Variation

**FIGURE 1:**
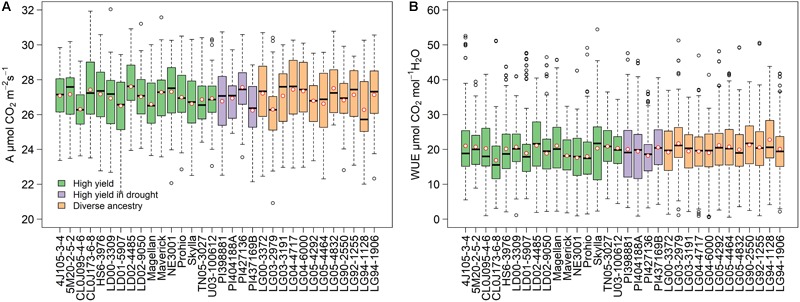
Phenotypic diversity for photosynthesis **(A)** and intrinsic water use efficiency **(B)** grouped by family in a soybean phenology-controlled panel. Three hundred and eighty-three cultivars, 12 cultivars per family, and three environments. Colors represent the type of population assigned to the parent when the SoyNAM panel was developed. Red circles denote the mean value, horizontal lines in the box indicate the median, dashed lines represent the minimum and maximum values, and empty circles correspond to outliers.

Phenotypic variation was observed for *A* and *WUE* (Figure 1). Photosynthetic rates ranged from 21.3 to 31.8 μmol CO_2_ m^-2^ s^-1^ with an overall mean of 27.0 μmol CO_2_ m^-2^ s^-1^ (Figure 1A). Statistically significant differences (*p* < 0.001) among environments were detected, with means of 26.9, 27.3, and 26.6 μmol CO_2_ m^-2^ s^-1^ for ACRE_2017, ACRE_2018, and RMN_2018, respectively (Supplementary Figures S6–S8). The ratio *A*/*gs*, also called intrinsic *WUE* (Condon et al., 2002; Gilbert et al., 2011; Blankenagel et al., 2018), showed an overall mean of 20 μmol CO_2_ mol^-1^ H_2_O (Figure 1C). Statistically significant differences (*p* < 0.001) among environments were detected, with means of 20.2, 17.1, and 22.7 μmol CO_2_ mol^-1^ H_2_O for ACRE_2017, ACRE_2018, and RMN_2018, respectively (Supplementary Figures S6–S8). We observed the lowest *WUE* in families CLOJ173-6-8, NE3001, and Prohio, with 16.9, 17.6, and 18.0 μmol CO_2_ mol^-1^ H_2_O, contrasting with LG94-1128, LG94-2979, and LG90-2550 with 22.8, 21.7, and 21.3 μmol CO_2_ mol^-1^ H_2_O, respectively.

### Genetic Architecture

**FIGURE 2:**
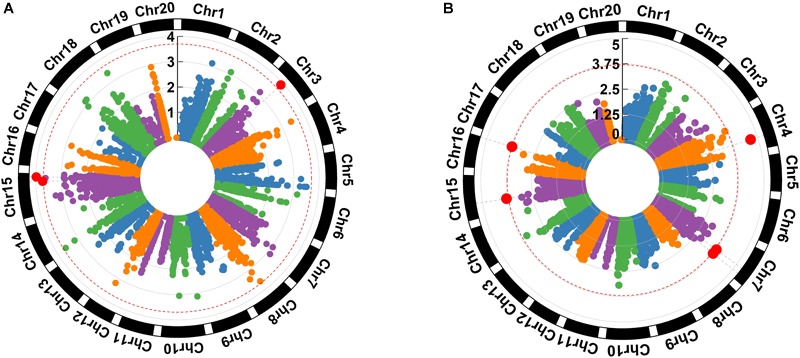
Genetic architecture for photosynthesis **(A)** and intrinsic water use efficiency **(B)** in a phenology-controlled soybean panel. Three hundred and eighty-three cultivars and three environments. Chr indicates the chromosome, the red dashed circle represents the threshold expressed as –log *p*-value, and red dots correspond to the significant SNPs.

The genome-wide association analysis identified SNPs and genomic regions associated with *A* and *WUE* (Figure 2). Associations were found between photosynthesis and one SNP located on chromosome 3 and two SNPs on chromosome 15. Intrinsic *WUE*, in turn, was associated with two SNPs on chromosome 7 and single SNPs on chromosomes 4, 15, and 16. All SNPs detected were located in euchromatic regions (Song et al., 2016) except for the SNPs for photosynthesis and intrinsic *WUE* located on chromosome 3 and 16, respectively (Table 2).

**Table 2:** Significant single nucleotide polymorphism (SNP) associated with photosynthesis (*A*) and intrinsic water use efficiency (*WUE*) in soybean.

Trait	Chr	Position	SNP	LOD	σ ^2^	Gene	Annotation
*A*	3	16015499	A/G	2.7	1.8	NA	NA
	15	46787588	C/T	2.8	4.3	Glyma.15g245300	Cytochrome P450 family member
	15	41081388	C/T	2.5	2.9	Glyma.15g225000	NADH oxidoreductase-related
	4	33984084	C/A	3.4	5.2	Glyma.04g152000	Alpha carbonic anhydrase 6
	7	29855554	C/T	2.6	5.4	Glyma.07g172000	Trehalose-6-phosphate synthase
*WUE*	7	37317294	T/C	2.6	5.2	Glyma.07g204400	Glucosyl transferase
	15	17653104	T/C	2.6	5.3	Glyma.15g182600	Glucosyl transferase – sucrose synthase
	16	36805830	C/T	2.5	5.5	NA	NA

*Chr, chromosome; LOD, logarithm of the odds; σ^*2*^, variance explained by the SNP.*

### Genetic, Additive Variances, and Genetic Correlations

The proportion of the variance explained by genetics was 21% for *A* and 33% for *WUE* (Table 3). Repeatability, or heritability on an entry mean basis, reached 0.44 for *A* and 0.60 for *WUE*. Additive genetic effects calculated through the kinship or “*K*” matrices, generated separately using SNPs with -log *p*-values higher than 0.0, 0.5, and 1.0, were able to account for 82–95% of the genetic variance estimated (Table 3). Although smaller kinship matrices built with SNPs with -log *p*-values thresholds of 1.5 and 2.0 are not able to explain the genetic variance completely, they were still successful capturing ∼87 and ∼65% of the total genetic variance, respectively. Remarkably high genetic correlations between grain yield and photosynthesis was observed with a value of 0.80.

**Table 3:** Broad sense heritability on plot basis (*H* plot), entry basis *(H* entry), and narrow (*h^2^*) sense heritability as function of the SNPs data set considered in soybean.

Trait	*H* plot	*H* entry	*h^2^* SNP > 0	*h^2^* SNP > 0.5	*h^2^* SNP > 1.0	*h^2^* SNP > 1.5	*h^2^* SNP > 2.0
*A*	0.21	0.44	0.87	0.92	0.95	0.85	0.60
*WUE*	0.33	0.60	0.82	0.90	0.95	0.89	0.70

*SNPs were filtered based on the -log *p*-value from a genome wide association study. Photosynthesis (*A*) and intrinsic water use efficiency (*WUE*).*

### Genomic Prediction

**FIGURE 3:**
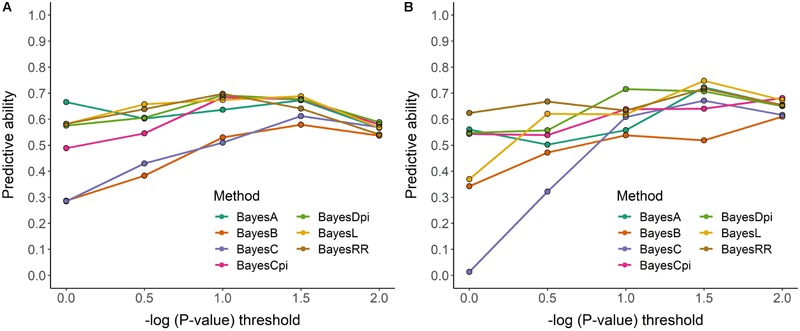
Genomic prediction performance based of five-fold cross-validation of Markov Chain Monte Carlo (MCMC) methods for photosynthesis **(A)** and intrinsic water use efficiency **(B)** in a phenology-controlled soybean panel. Three hundred and eighty-three cultivars and three environments.

When SNPs in the interval -log *p*-value > 0.0 to -log *p*-value > 1.0 were used, genome regressions via MCMC (Figure 3) showed stable predictive ability, described as the correlation coefficients (*R*) between predicted and observed values. Maximum correlation for *A* (Figure 3A) and *WUE* (Figure 3B) of 0.70 and 0.74 were determined. Regardless the threshold considered to select the SNPs, *BayesL* presented the highest pooled predictive ability for *A* with 0.63, while *BayesDPi* showed the best performance for *WUE* with predictive ability of 0.64. Although their general performance improved when the SNP data set became smaller (-log *p*-value > 1.5 and –log *p*-value > 2.0), the variable selection methods *BayesB* and *BayesC* displayed the lowest correlation through the different SNPs data sets. *BayesB* reported 22–27% less predictive ability compared with the best methods, while *BayesC* exhibited 24–30% lower correlation. Similar results were also found when the same set of models was fitted through EM regressions (Supplementary Figure S9). In this case, a maximum correlation of 0.67 and 0.70 were observed for *A* and *WUE*, respectively.

## Discussion

### Phenotypic Variation

For photosynthesis, or *A*, values of 25–35 μmol CO_2_ m^-2^ s^-1^ were reported for soybeans in field (Gordon et al., 1982) and greenhouse conditions (Hay et al., 2017). These values are comparable to our observations considering the potential limitations offered by the device used decades ago. Comparison at the family level between the mean rate and the maximum *A* attainable indicates a potential increase of at least 20%. Intrinsic *WUE* has been less studied than *A*, but this research reveals that there is also natural diversity to be exploited through breeding programs. Water use is considered a limiting factors in the modern soybean production (Specht et al., 1999) accounting for until 30% of the yield gap (Grassini et al., 2015). A positive and significant correlation (0.78) between transpiration, the main source of water loss in plants, and yield was documented in Chinese cultivars (Liu et al., 2012).

Although enhanced transpiration and *WUE* have been set as functional target in new soybean cultivars (Sloane et al., 1990; Manavalan et al., 2009; Miladinović et al., 2015), achieving substantial progress in yield minimizing the water consume is challenging. A study in china using materials of 82 year of soybean breeding found an unbalance improvement between *E* and *A* with increases in transpiration rate of ∼58% while photosynthesis barely reached ∼18% (Liu et al., 2012). Though these authors conclude that the biggest cost of producing high yielding soybean cultivars is the augmented water consume, our results show that doubling the intrinsic *WUE* (*A*/*gs*) might be possible. Intrinsic *WUE* here documented is similar to previous reports of Gilbert et al. (2011) and Bunce (2016) in soybean and it is also in the range of other C3 species (Medrano et al., 2015). Comparing the overall mean value (20 μmol CO_2_ mol^-1^ H_2_O) with the maximum attainable (54.4 μmol CO_2_ mol^-1^ H_2_O), it is hypothesized until 34 μmol CO_2_ (1.5 mg CO_2_) extras can be fixed with the same rate of H_2_O efflux through the stomata. Better understanding in genetic basis of stomatal control seems to play a key role to reduce the gap between actual and potential *WUE* in soybean (Gilbert et al., 2011).

### Genetic Architecture

Although genetic architecture for these physiological processes has been a topic underexplored, the current study found SNPs potentially associated with the traits. *A* is closely linked to genes encoding for protein members of the cytochrome P450 family and NADH oxidoreductase. Cytochrome proteins catalyze the oxidation of diverse substrates using oxygen and NAD(P)H (Xu et al., 2010). In plants, they are functionally active transporting electrons and molecular oxygen generated during the photosynthesis (Burow et al., 2016). Though there were no QTLs associated with photosynthesis or carbon fixation previously reported for chromosome 15 (Grant et al., 2010), there is a reasonable background to hypothesize the relationship between photosynthesis and cytochrome. In cyanobacterium, for instance, an improved performance in *A* via increased electron transport rate and ATP production was promoted as consequence of doubling the activity of the cytochrome protein CYP1A1 (Berepiki et al., 2018). Induction of genes associated with these proteins are also reported when atrazine and bentazon, herbicides inhibitor of photosynthesis, are applied in soybean (Zhu et al., 2009). Likewise, enhanced tolerance to linuron and chlortoluron, herbicides also inhibitor of photosynthesis, are documented when the expression of the cytochrome P450 protein CYP76B1 in tobacco and Arabidopsis (Didierjean et al., 2002) and CYP71A10 in soybean (Siminszky et al., 1999) were carried out. QTLs associated with photosynthesis under light saturation had been already reported for chromosome 10 and 16 (Vieira et al., 2006).

The other association found on the chromosome 15 was linked to a gene encoding for NADH oxidoreductase-related. This type of protein catalyzes the oxidation of NADH and the reduction of other compound (Moparthi and Hägerhäll, 2011). The most common enzyme in this group is the NADH-ubiquinone oxidoreductase, the largest enzyme in the mitochondrial respiratory chain (Cardol, 2011). Respiration is the natural complementary process of photosynthesis and their balance defines the net photosynthetic rate, the parameter measure by the infrared gas exchange equipment. Then, it is hypothesized NADH oxidoreductase influences photosynthesis through regulation of oxidation-reduction rates presumably during the respiration. The association found on the chromosome 3 had no previous annotation or QTLs reported into the standard linkage block (Wen et al., 2015) which might be explained by its location in a heterochromatic pericentromeric region (Song et al., 2016). The location of this SNP in a region with low recombination rate implies that its LD is larger than the value reported for whole the chromosome, extending the association to wider areas. Large differences in LD pericentromeric regions and arm regions been confirmed for soybean (Shu et al., 2015).

Intrinsic *WUE* was associated with regions on the chromosome 4, 7, 15, and 16 (Table 2). QTLs potentially associated with *E* and *WUE* had been previously reported on the chromosome 4 (Kaler et al., 2017) but none of them overlap with the QTL documented here. The closers QTLs already reported correspond to *WUE 2-g11* and *WUE 2-g12*, whose annotated genes link to Glyma04g39850 (Integral membrane protein DUF6) and Glyma04g41150 (RNA recognition motif. a.k.a. RRM, RBD, or RNP domain) (Grant et al., 2010). A carbonic anhydrase is proposed considering its role in the interconversion of CO_2_ to HCO_3_^-^, a fundamental step in the carbon dioxide movement in aqueous medium type leaf cytosol (DiMario et al., 2017). In C3 plants like soybean, carbonic anhydrase can increase the carbon fixation through raising the internal CO_2_ concentration in the chloroplast which reduces the photorespiration (Ganai, 2017).

The SNP associated with *WUE* that we observed on chromosome 7 at the position 29855554 bp is associated with a trehalose-6-phosphate synthase. Trehalose is a disaccharide present in bacteria, fungi, and invertebrates linked with abiotic stress tolerance given its role as energy source, compatible osmolyte, and protein/membrane stabilizer (Garg et al., 2002; Iordachescu and Imai, 2008). Trehalose-6-phosphate, an intermediate compound, acts as sucrose availability sensor, while its overexpression increases stomatal guard cell sensitivity, enabling fast stomata closing under drought stress (Delorge et al., 2014). The SNP associated with *WUE* at position 37317294 bp that we observed on chromosome 7, as well as the SNP on chromosome 15, were associated with genes expressing glucosyl transferase; a member of a large family of enzymes in charge of transferring sugar moieties between molecules (Lairson et al., 2008). Glucosyl transferases type uridine diphosphate UDP are involved in osmotic, salt, and drought stress response in cotton (Tai et al., 2008), Arabidopsis (Li et al., 2015; Li P. et al., 2017; Zhang et al., 2016), and tobacco (Sun et al., 2013). Our observed QTL on chromosome 7 do not overlap with drought tolerance QTLs documented previously on this chromosome as *drought susceptibility index 6* (Du et al., 2009). The SNP we observed on chromosome 15 is also linked with sucrose synthase enzyme. In soybean nodules, a dramatic reduction in sucrose synthase activity was reported by González et al. (1995) as a consequence of water deprivation; the authors of that study proposed that this enzyme played a role in nitrogen fixation, which would be central to maintaining high assimilation rates under drought conditions. A QTL for pubescence density is also already reported in the same linkage block on chromosome 15 (Grant et al., 2010). Leaf pubescence density may decrease leaf temperature and restrict transpiration water loss via increased leaf boundary layer resistance (Manavalan et al., 2009).

### Heritability and Genetic Correlations

Heritability for the traits considered in this work is moderate to low (Holland et al., 2010). Phenotyping gas exchange implies challenges since these processes are highly influenced by environmental factors such as light, temperature, nitrogen, and water status among others (Taiz et al., 2014). Although the equipment and protocol implemented to phenotype these traits attempted to control most of these external variables, moderate to high influence of factors beyond genetic was captured. Genetic effects explain 44 and 60% of changes in the phenotypic values for *A* and *WUE*, respectively. Heritability in entry mean bases for *A* computed here is similar to the 41% previously reported in soybean (Harrison et al., 1981). When kinship matrices estimated with the SNPs associated with the specific trait were considered, additive or transmissible effects accounted by 82–95% of the genetic effect. The drastic reduction in the number of SNPs used to estimate the kinship matrix limits the ability to capture the genetic resemblance between individuals. The additive effect can be captured with a considerable less number of SNPs, as long as these SNPs show certain level of association with the trait (Table 3). However, matrices excessively reduced are not able to capture the additive effects completely. Including an extension of the additive matrix through an extra matrix additive-by-additive (epistasis) was attempted (data not shown) for scenarios with reduced number of SNPs (-log *p*-value 1.5 and -log *p*-value 2.0). The additive-by-additive matrix improved the heritability in the range of 11–34% when the number of SNPs was the lowest (-log *p*-value 2.0) but it did not show substantial improvement when -log *p*-value > 1.5 matrix was considered. Introducing epistatic effects might help to explain genetic effects when the number of markers is reduced. The fact heritability is relatively constant in the interval -log *p*-value > 0.0 to -log *p*-value > 1.0 but decreases when data set becomes smaller aligns with previous reports for flowering date, height and nodule number in alfalfa (Stanton-Geddes et al., 2013). In this case, authors reported similar *h^2^* calculated through SNPs data sets with size ranged between 25,000 and 5 million but comparably lower *h^2^* when 2,500 and 25 SNPs data sets were used.

We observed a lack of phenotypic correlation between *A* and *GY* (-0.02) that agrees with previous reports summarized by Long et al. (2006); however, the design of our study allowed us to also calculate the genetic correlations, which was positive (0.80). This observation indicates strong non-genetic factors influencing assimilation rate. A high genetic correlation between traits predicts potential outcomes of selection through indirect gains (Searle, 1978) and our results indicate selection to increase *A* may positively affect grain yield.

### Genomic Prediction

The complexity of field measurements, the limited variance explained by significant SNPs, and the moderate to low heritability for these traits, suggest genomic prediction-selection as suitable methodology to approach their breeding (Desta and Ortiz, 2014; Xavier et al., 2016). This work indicates this approach is feasible since predictability through MCMC or Estimation Maximization yields moderate to high correlation coefficients. The correlation found here are lower that the values reported for oil and protein (0.92), but comparable with yield (0.60–0.79), yield component and morph-physiological parameters like plant height, number of reproductive nods and days to maturity in soybean (Jarquin et al., 2016; Xavier et al., 2016). Although limiting the number of SNPs influences the predictive ability especially when -log *p*-value is higher than 1.5 and 2.0, the reduction in predictive ability for none of the traits was higher than 20%. The use of selected sets of SNPs for genomic prediction was previously reported in crops and animals. In eucalyptus, for instance, similar predictive ability between large (∼14,000–20,000) and reduced (∼5,000–10,000) SNPs data sets was reported by Müller et al. (2017). In Brahman cattle, Li et al. (2018) working with body weight, demonstrated that data subsets of 3,000 SNPs selected through machine learning methods yielded similar prediction accuracy than full genome prediction through 38,082 SNPs. Genomic selection with low density SNPs has also reported other benefits in breeding. Raoul et al. (2017), for instance, improved the genetic gain and better control the inbreeding in sheep using a SNP data set ≤ 1,000 SNPs. Besides technical advantages, reduced data sets also decrease the computational time to fit the model up to 50% (Xavier et al., 2017b). Genome prediction through relatively small number of SNPs (∼1,000–2,000) moderately associated with the trait (-log *p*-value ≥ 1.0) is an appropriate and consistency approach to predict photosynthesis and intrinsic *WUE* across most of the methods considered. Although the implementation of different methods for genome regression in soybean yield and protein did not show significant improvement (Duhnen et al., 2017), the methods ML, BLASSO and *BayesDPi* showed a better performance for the traits evaluated here. The fact *Bayes B* and *BayesC* had shown lower predictive ability suggests that variable selection did not favor the predictive ability for these physiological traits. Contrarily, a quality control based on the significance of the genome-wide association results was beneficial.

## Conclusion

The existence of natural diversity and a preliminary genetic architecture for photosynthesis and intrinsic *WUE* indicate these traits can be improved through breeding strategies. New technologies like genomic selection-prediction arises as valuable approach to overcome the phenotyping bottleneck in gas exchange. Pre-selecting SNPs for genomic prediction modeling based on the significance of associations can benefit the predictive ability. Improving photosynthesis through breeding techniques is a viable strategy to increase yield in soybean, considering their high positive genetic correlation.

## Data Availability

The datasets generated for this study can be found in SoyBase.org, https://www.soybase.org/SoyNAM/.

## Author Contributions

ML and KR conceived and designed the experiments. ML and AX conducted the data analysis and interpretation. ML wrote and edited the manuscript. KR coordinated and supervised the research, and acquired funding.

## Conflict of Interest Statement

The authors declare that the research was conducted in the absence of any commercial or financial relationships that could be construed as a potential conflict of interest.

## Footnotes

1 www.soybase.org/SoyNAM

## Supplementary Material

FIGURE S1 Comparative for yield and maturity between the phenology-controlled panel and the full Soy-NAM panel.

FIGURE S2 Light response curve for random selected soybean cultivars to determine the stable phase in photosynthesis.

FIGURE S3 Histogram of distribution for photosynthesis **(A)** and intrinsic water use efficiency **(B)**. Three hundred and eighty-three cultivars and three environments.

FIGURE S4 Diurnal dynamic for photosynthesis **(A)** and intrinsic water use efficiency **(B)**. Three hundred and eighty-three cultivars and three environments.

FIGURE S5 Descriptive information about SNPs. Principal components discrimination of the 32 families considered based on genomic information (SNP) **(A)**. Original allele frequency of the 50K projected SNP for the phenology-controlled soybean panel **(B)**.

FIGURE S6 Phenotypic variation for photosynthesis **(A)** and intrinsic water use efficiency **(B)** in location ACRE_2017. Three hundred and two cultivars and three environments. Colors represents the type of population assigned to the parent when the SoyNAM panel was developed. Red circles denotes the mean value. Red circles denote the mean value, horizontal lines in the box indicate the median, dashed lines represent the minimum and maximum values and empty circles correspond to outliers.

FIGURE S7 Phenotypic variation for photosynthesis **(A)** and intrinsic water use efficiency **(B)** in location ACRE_2018. Three hundred and eighty-two cultivars and three environments. Colors represents the type of population assigned to the parent when the SoyNAM panel was developed. Red circles denotes the mean value. Red circles denote the mean value, horizontal lines in the box indicate the median, dashed lines represent the minimum and maximum values and empty circles correspond to outliers.

FIGURE S8 Phenotypic variation for photosynthesis **(A)** and intrinsic water use efficiency **(B)** in location RMN_2018. Three hundred and sixty-eight cultivars and three environments. Colors represents the type of population assigned to the parent when the SoyNAM panel was developed. Red circles denotes the mean value. Red circles denote the mean value, horizontal lines in the box indicate the median, dashed lines represent the minimum and maximum values and empty circles correspond to outliers.

FIGURE S9 Genomic prediction performance based of 5-fold cross-validation of expectation maximization (EM) methods for photosynthesis **(A)** and intrinsic water use efficiency **(B)** in a phenology-controlled soybean panel. Three hundred and eighty-three cultivars and three environments.

Table S1 Name, class and program of origin for the 32 families evaluated.

Table S2 List of recombinant inbred lines evaluated including family and class.

Table S3 Soil fertility information based on soil analyses reports.

Table S4 Water balance for the location ACRE_2017.

Table S5 Water balance for the location ACRE_2018.

Table S6 Water balance for the location RMN_2018.
