# Supplementary figures and images for: Phenotypic Variation and Genetic Architecture for Photosynthesis and Water Use Efficiency in Soybean (Glycine max L. Merr)
Source: Front Plant Sci. 2019 May 24;10:680. doi: 10.3389/fpls.2019.00680 (PMC6543851; doi:10.3389/fpls.2019.00680)

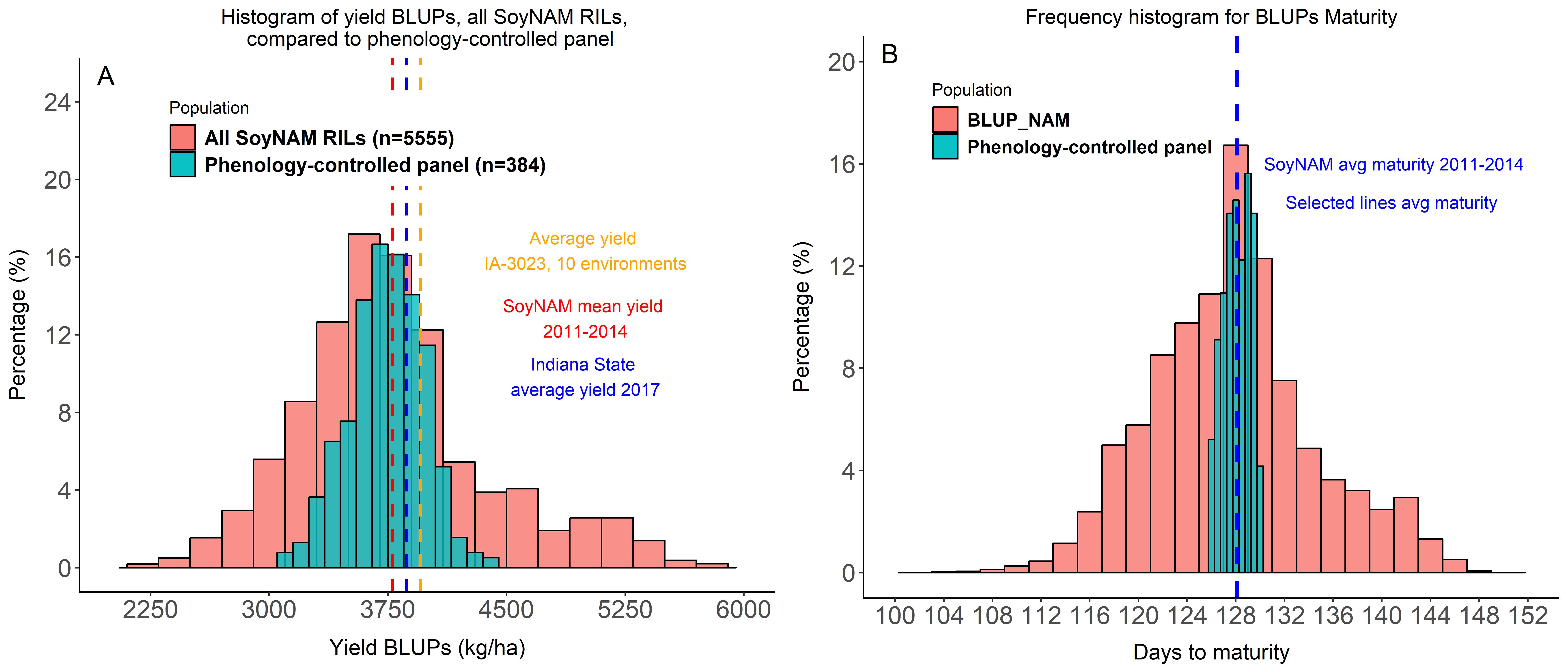

Supplement: FIGURE S1 — Comparative for yield and maturity between the phenology-controlled panel and the full Soy-NAM panel. [file Image_1.JPEG]

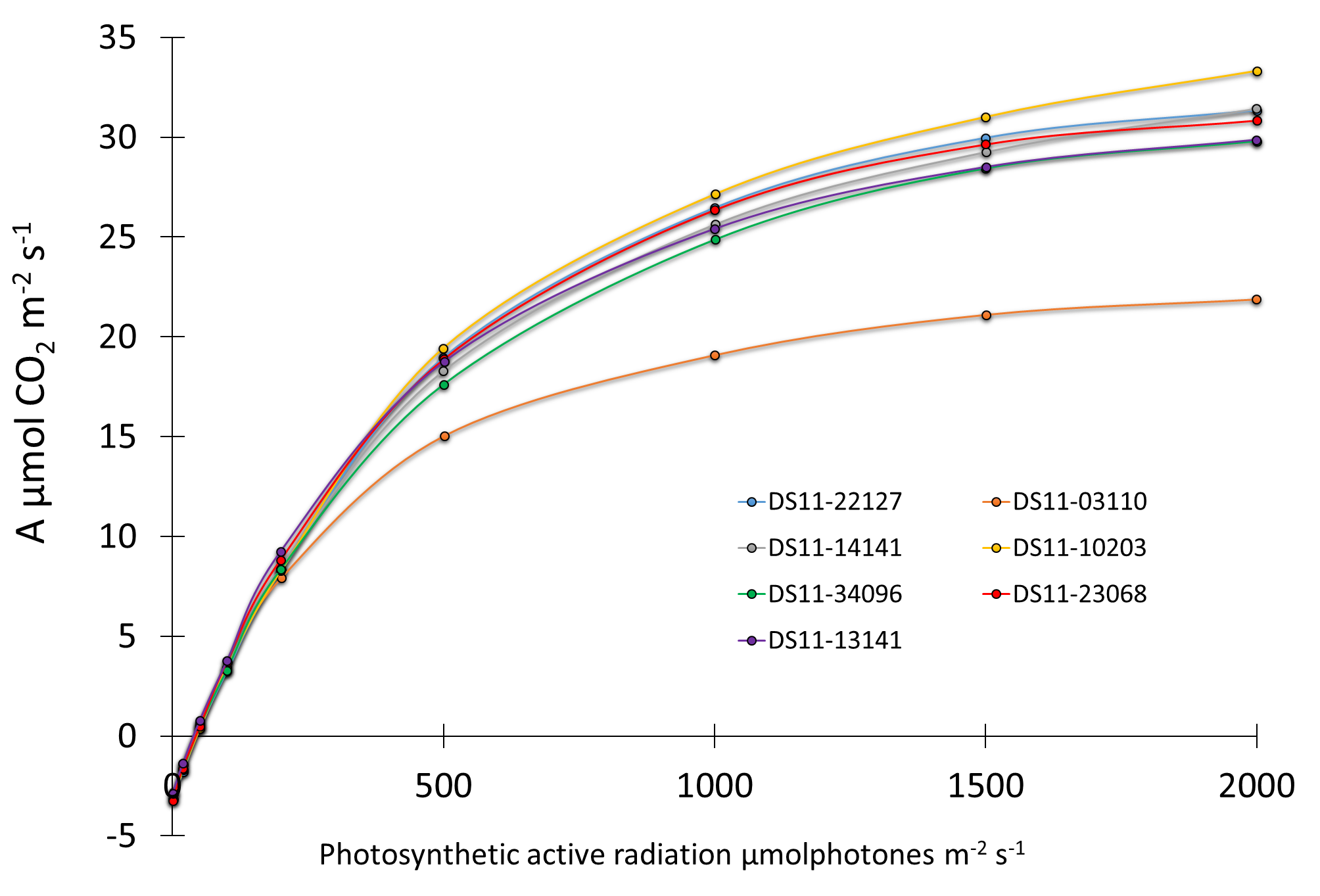

Supplement: FIGURE S2 — Light response curve for random selected soybean cultivars to determine the stable phase in photosynthesis. [file Image_2.PNG]

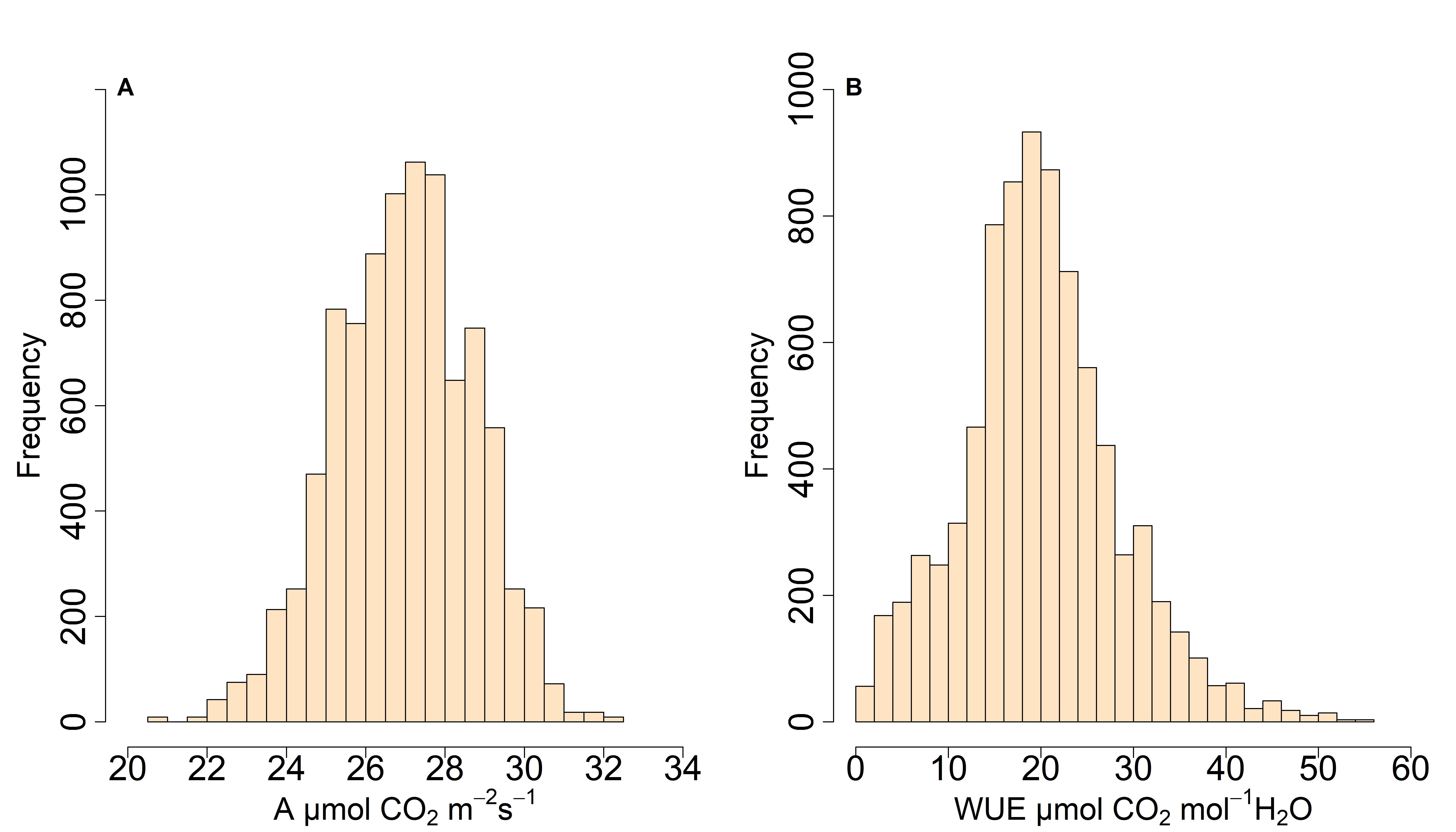

Supplement: FIGURE S3 — Histogram of distribution for photosynthesis (A) and intrinsic water use efficiency (B). Three hundred and eighty-three cultivars and three environments. [file Image_3.JPEG]

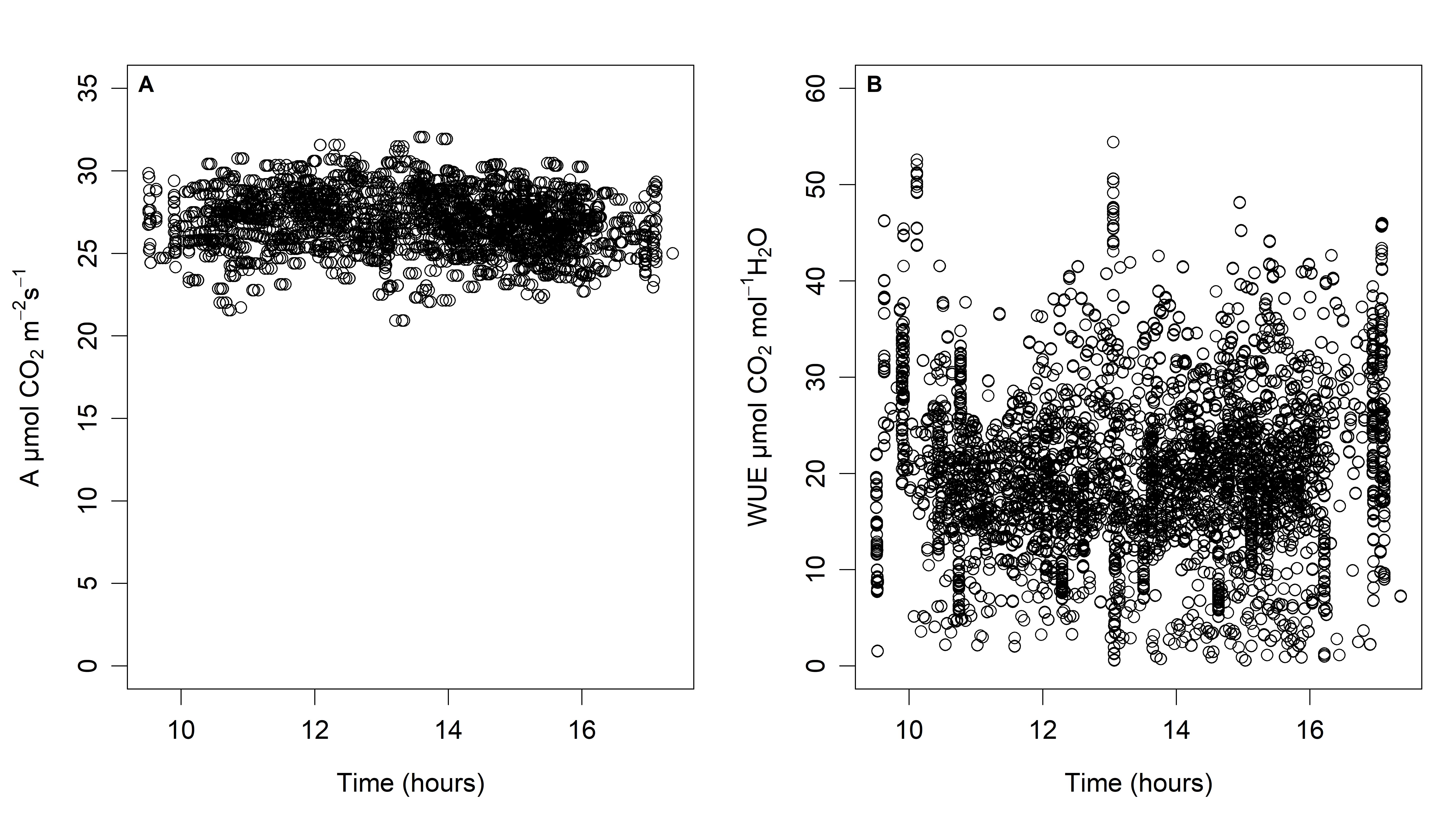

Supplement: FIGURE S4 — Diurnal dynamic for photosynthesis (A) and intrinsic water use efficiency (B). Three hundred and eighty-three cultivars and three environments. [file Image_4.JPEG]

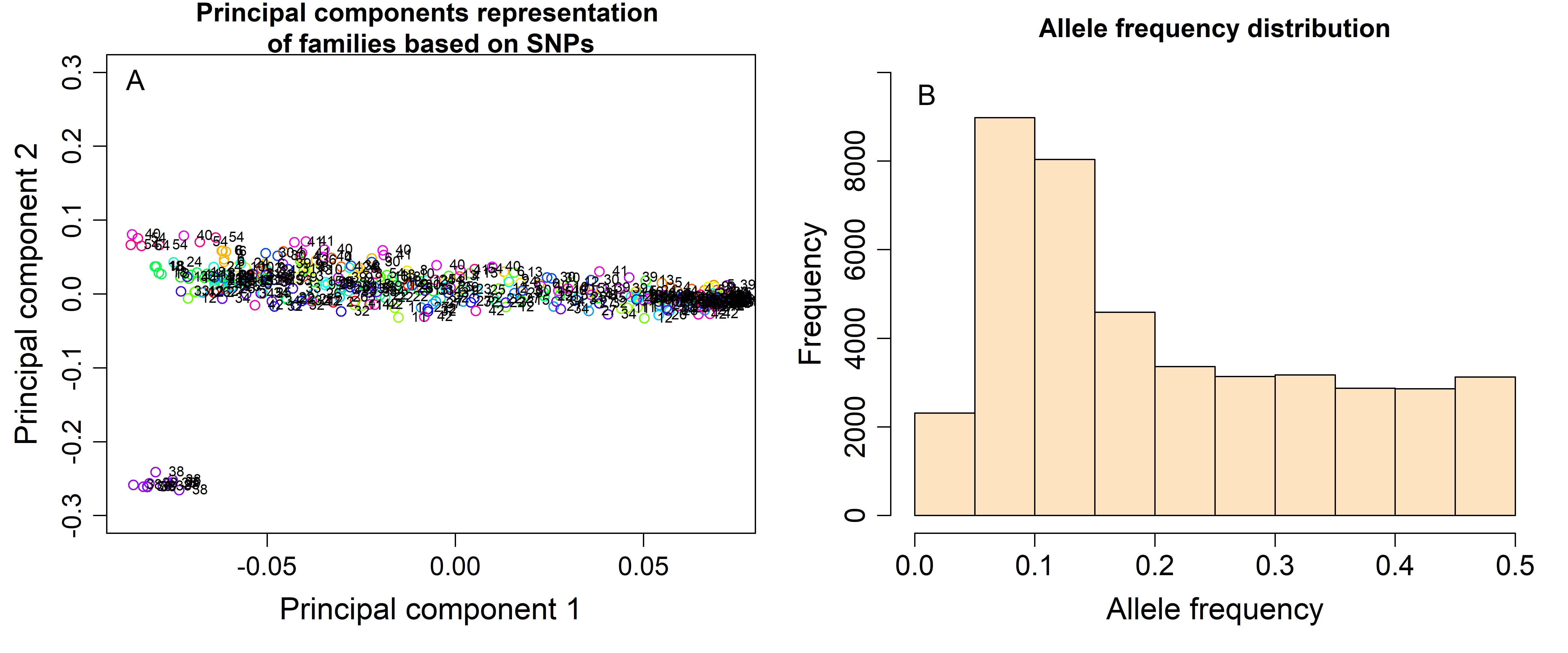

Supplement: FIGURE S5 — Descriptive information about SNPs. Principal components discrimination of the 32 families considered based on genomic information (SNP) (A). Original allele frequency of the 50K projected SNP for the phenology-controlled soybean panel (B). [file Image_5.JPEG]

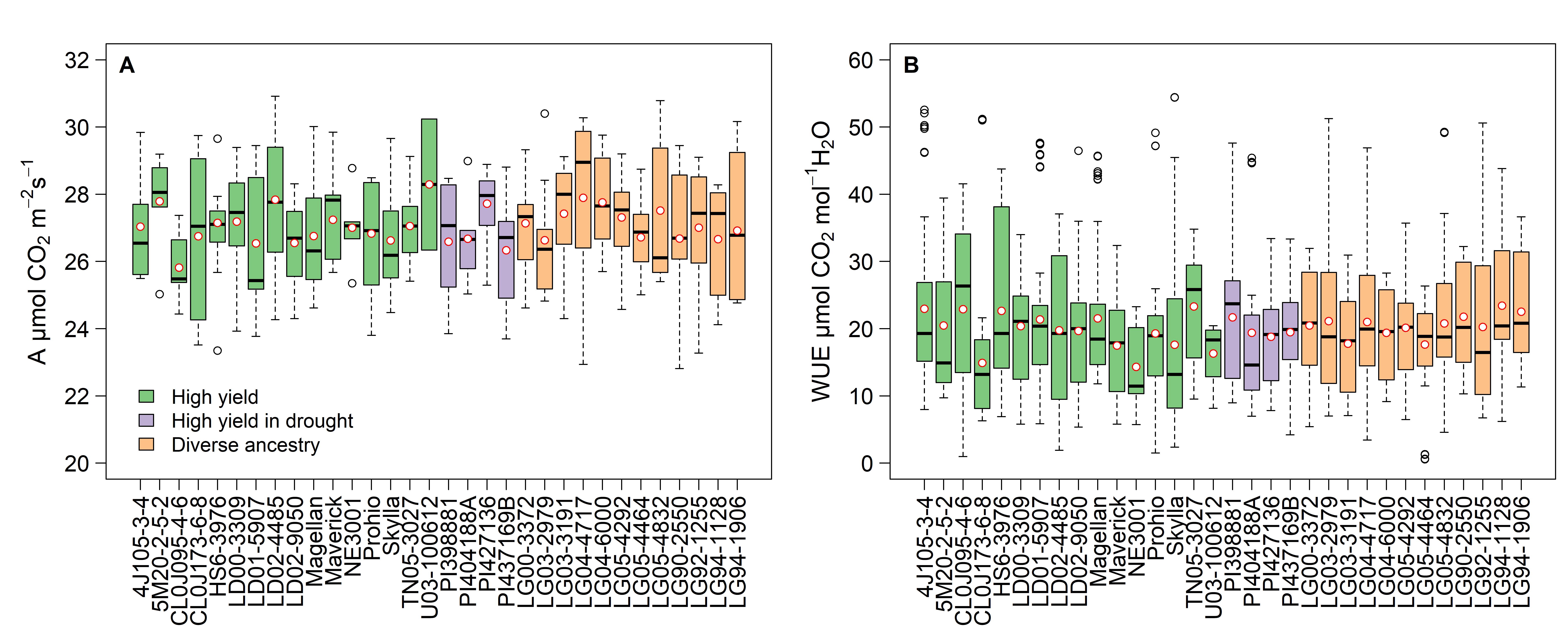

Supplement: FIGURE S6 — Phenotypic variation for photosynthesis (A) and intrinsic water use efficiency (B) in location ACRE_2017. Three hundred and two cultivars and three environments. Colors represents the type of population assigned to the parent when the SoyNAM panel was developed. Red circles denotes the mean value. Red circles denote the mean value, horizontal lines in the box indicate the median, dashed lines represent the minimum and maximum values and empty circles correspond to outliers. [file Image_6.JPEG]

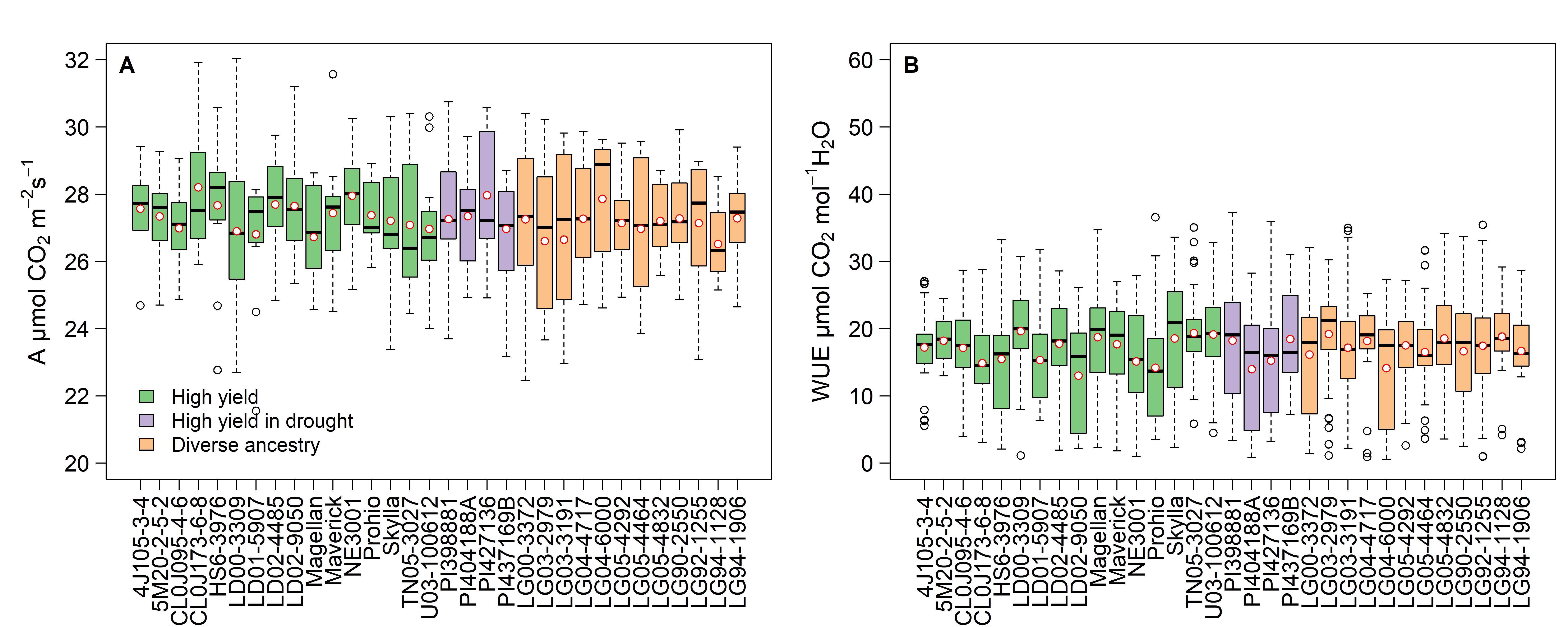

Supplement: FIGURE S7 — Phenotypic variation for photosynthesis (A) and intrinsic water use efficiency (B) in location ACRE_2018. Three hundred and eighty-two cultivars and three environments. Colors represents the type of population assigned to the parent when the SoyNAM panel was developed. Red circles denotes the mean value. Red circles denote the mean value, horizontal lines in the box indicate the median, dashed lines represent the minimum and maximum values and empty circles correspond to outliers. [file Image_7.JPEG]

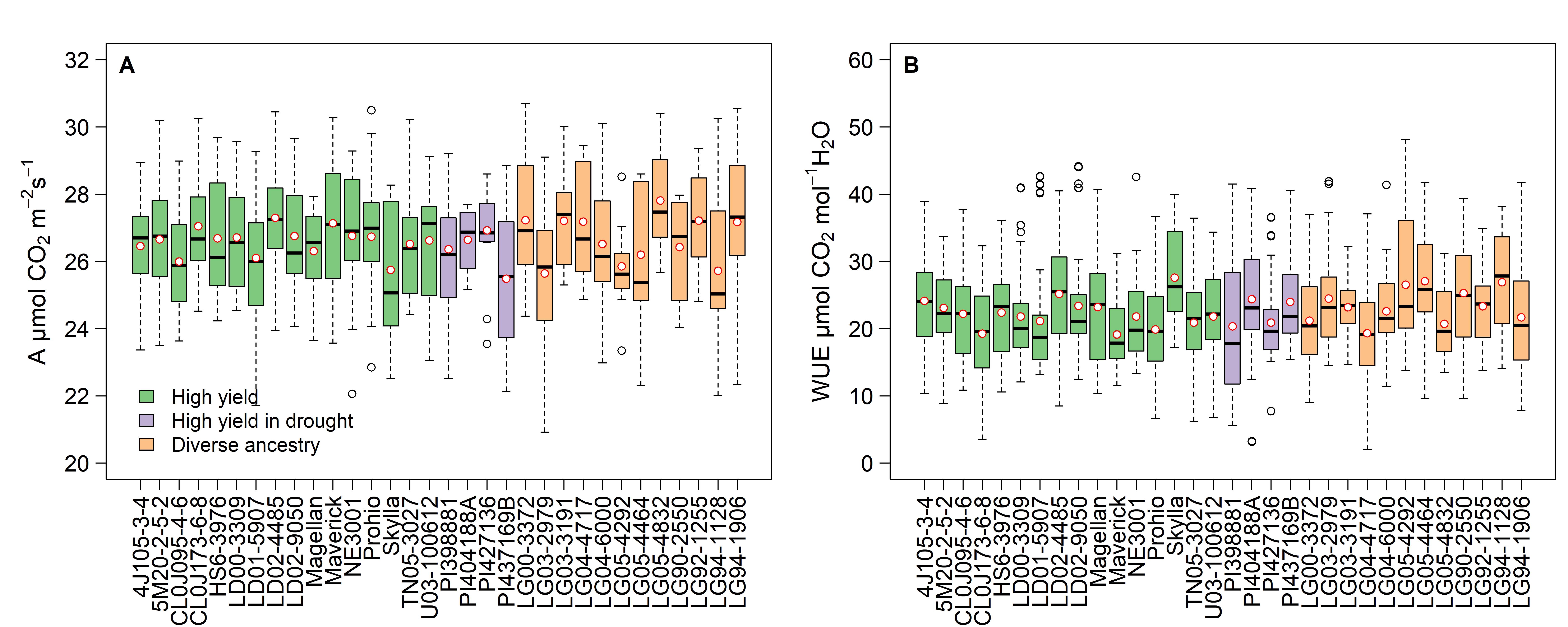

Supplement: FIGURE S8 — Phenotypic variation for photosynthesis (A) and intrinsic water use efficiency (B) in location RMN_2018. Three hundred and sixty-eight cultivars and three environments. Colors represents the type of population assigned to the parent when the SoyNAM panel was developed. Red circles denotes the mean value. Red circles denote the mean value, horizontal lines in the box indicate the median, dashed lines represent the minimum and maximum values and empty circles correspond to outliers. [file Image_8.JPEG]

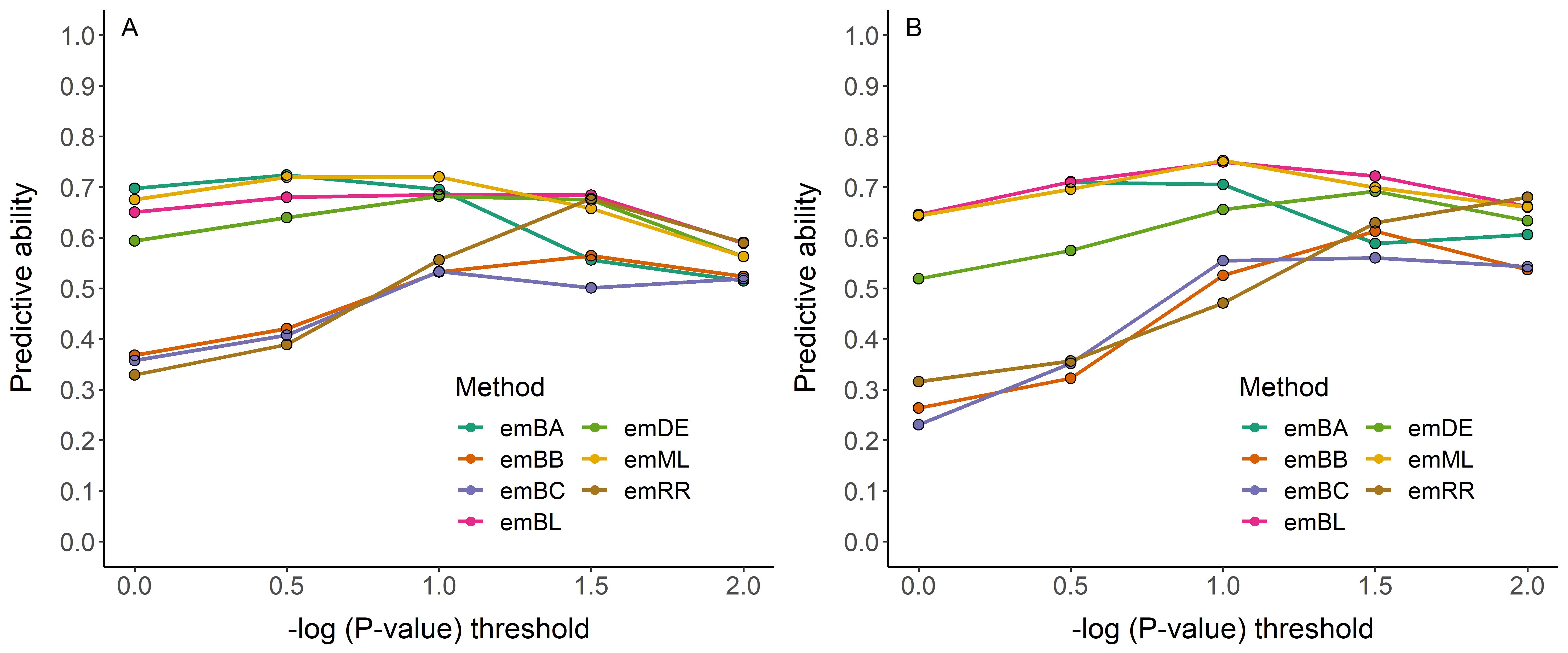

Supplement: FIGURE S9 — Genomic prediction performance based of 5-fold cross-validation of expectation maximization (EM) methods for photosynthesis (A) and intrinsic water use efficiency (B) in a phenology-controlled soybean panel. Three hundred and eighty-three cultivars and three environments. [file Image_9.JPEG]
